# Improving isopropanol tolerance and production of *Clostridium beijerinckii* DSM 6423 by random mutagenesis and genome shuffling

**DOI:** 10.1007/s00253-016-7302-5

**Published:** 2016-02-06

**Authors:** H. Máté de Gérando, F. Fayolle-Guichard, L. Rudant, S. K. Millah, F. Monot, Nicolas Lopes Ferreira, A. M. López-Contreras

**Affiliations:** 1 Food and Biobased Research Wageningen UR, Wageningen, the Netherlands; 2 Biotechnology Department, IFP Energies nouvelles, Rueil-Malmaison, France

**Keywords:** *Clostridium beijerinckii*, Mutagenesis, Genome shuffling, Isopropanol tolerance, IBE fermentation

## Abstract

Random mutagenesis and genome shuffling was applied to improve solvent tolerance and isopropanol/butanol/ethanol (IBE) production in the strictly anaerobic bacteria *Clostridium beijerinckii* DSM 6423. Following chemical mutagenesis with N-methyl-N-nitro-N-nitrosoguanidine (NTG), screening of putatively improved strains was done by submitting the mutants to toxic levels of inhibitory chemicals or by screening for their tolerance to isopropanol (>35 g/L). Suicide substrates, such as ethyl or methyl bromobutyrate or alcohol dehydrogenase inhibitors like allyl alcohol, were tested and, finally, 36 mutants were isolated. The fermentation profiles of these NTG mutant strains were characterized, and the best performing mutants were used for consecutive rounds of genome shuffling. Screening of strains with further enhancement in isopropanol tolerance at each recursive shuffling step was then used to spot additionally improved strains. Three highly tolerant strains were finally isolated and able to withstand up to 50 g/L isopropanol on plates. Even if increased tolerance to the desired end product was not always accompanied by higher production capabilities, some shuffled strains showed increased solvent titers compared to the parental strains and the original *C. beijerinckii* DSM 6423. This study confirms the efficiency of genome shuffling to generate improved strains toward a desired phenotype such as alcohol tolerance. This tool also offers the possibility of obtaining improved strains of *Clostridium* species for which targeted genetic engineering approaches have not been described yet.

## Introduction

With the growing concern of petroleum shortage and the negative environmental impact of fossil resource extraction and transformation, there is a renewed worldwide interest for environmentally friendly ways to produce fuels and chemicals (Chen et al. 2013). Alcohols are natural major end products of some microbial fermentations. The acetone/butanol/ethanol (ABE) or the isopropanol/butanol/ethanol (IBE) fermentation processes have a long industrial history and have been used almost uninterruptedly from the 1910s on when the first plants were built in the UK (Jones and Woods 1986). These were the main processes used for the production of butanol at an industrial scale and started to be replaced by the emerging petrochemical industry from the 1960s onward due to economic considerations (López-Contreras et al. 2012). Isopropyl alcohol or isopropanol (IpOH) is currently manufactured from propylene, a by-product of oil refining, either by an indirect or by a catalytic hydration process. It is a valuable product mainly used as a solvent in inks and cosmetics or as an antiseptic agent. It is nowadays obtained by petrochemical ways, but a dehydratation procedure offers the possibility to reverse the chemical process and obtain propylene from IpOH instead. Propylene is the second most important starting product in the petrochemical industry and is industrially produced exclusively from fossil fuels. IpOH obtained by fermentation of renewable resources could therefore be an interesting “biosourced” starting product and substitute to industrial propylene production. Some clostridial species are solventogenic, anaerobic, Gram-positive bacteria and are therefore known for their capability to produce ABE or IBE. *Clostridium acetobutylicum* strains, which are ABE producers, constitute the most widely studied solvent-producing strains (Liao et al. 2015; Cho et al. 2015) and have been employed in large-scale productions of butanol (Berezina et al. 2012). In the case of isopropanol production, the IBE process has been also run on an industrial scale. The production of isopropanol together with butanol by fermentation was first reported in 1906 (López-Contreras et al. 2010). Several strains have been identified as natural IBE producers including *Clostridium beijerinckii* DSM 6423 (Chen and Hiu 1986). Continuous production of isopropanol and butanol has been studied using this strain (de Vrije et al. 2013; Survase et al. 2011). Moreover, several ABE-producing strains have been genetically engineered to produce isopropanol (Collas et al. 2012; Lee et al. 2012), but the successfully improved strains, showing high concentrations, yields, and ratios of isopropanol are still lacking a commercial application. Product inhibition due to solvent toxicity has been reported as a major drawback in solventogenic fermentations, especially butanol production in *C. acetobutylicum*. Therefore, higher resistance to solvent toxicity should theoretically allow the strain to produce higher levels of solvents (Heluane et al. 2011).

In order to improve the performances of solventogenic Clostridia, genetic approaches, such as mutagenesis and metabolic engineering, have been followed (Gong et al. 2009; Leja et al. 2011; Patnaik 2008). Metabolic engineering approaches have been mostly targeted toward improved product yields (Lütke-Eversloh 2014), in most cases butanol (Schiel-Bengelsdorf et al. 2013). These approaches have resulted in interesting new strains; however, limited success was achieved toward increasing product tolerance (Alsaker et al. 2010). Because product tolerance is a complex mechanism where many pathways are involved, mutagenesis has been used to generate butanol-tolerant strains, (Hermann and Fayolle 1985). Li and co-workers describe recently the use of mutagenesis to generate mutants with an improved ABE production by up to 46 % compared to the wild-type strain (Li et al. 2013).

In this study, we aimed at improving isopropanol production by the natural producer *C. beijerinckii* DSM 6423 using a genome shuffling approach. There is no publicly available complete genomic sequence of this strain to work with and no genetic tools described. Therefore, a randomized genome shuffling approach was applied to achieve higher isopropanol titers and productivity. Genome shuffling allows the recombination of entire genomes as well as multi-parental crossing usually associated with conventional breeding. It was described as a process combining the advantages of multi-parental recombination of entire genomes and as an efficient method for the evolution of strains toward desirable phenotypes (Zhang et al. 2002). This method has been applied to the improvement of many production pathways in microorganisms, including ABE production by *C. acetobutylicum* CICC 8012 (Gao et al. 2012) or 1,3-propanediol production by *C. diolis* DSM 15410 (Otte et al. 2009). Here, we describe a combined approach of chemical mutagenesis and genome shuffling to improve the production of IBE by *C. beijerinckii* DSM 6423.

## Materials and methods

### Microorganisms, media, and culture conditions

*Clostridium beijerinckii* DSM 6423 (also classified as NRRL B593) is a laboratory strain originally obtained from the DSMZ collection. This strain was cultivated in modified CGM (mCGM) (Siemerink et al. 2011). The mCGM media used in this study contained the following, per liter: 5 g yeast extract, 0.75 g KH_2_PO_4_, 0.75 g K_2_HPO_4_, 0.4 g MgSO_4_·7H_2_O, 0.01 g MnSO_4_·H_2_O, 0.01 g FeSO_4_·7H_2_O, 1 g NaCl, 2 g asparagine, 2 g (NH_4_)_2_SO_4_, 0.125 g cysteine, and 12.5 g glucose. While mCGM agar medium was composed of, per liter, 1 g yeast extract, 2 g tryptone, 0.5 g KH_2_PO_4_, 1 g K_2_HPO_4_, 0.1 g MgSO_4_·7H_2_O, 0.01 g MnSO_4_·H_2_O, 0.015 g FeSO_4_·7H_2_O, 0.013 g CaCl_2_, 0.002 g CoCl_2_, 0.002 g ZnSO_4_, 2 g (NH_4_)_2_SO_4_, 50 g glucose, and 12 g microbial agar.

Gapes media were used for fermentation (liquid) or for sporulation (agar plates). The Gapes liquid medium contained the following, per liter: 5 g yeast extract, 1 g KH_2_PO_4_, 0.76 g K_2_HPO_4_, 3 g NH_4_ acetate, 1 g MgSO_4_·7H_2_O, 0.1 g FeSO_4_·7H_2_O, 0.1 g *p-*aminobenzoic acid, and 60 g glucose. The Gapes agar medium was composed of the following, per liter: 5 g yeast extract, 1 g KH_2_PO_4_, 0.61 g K_2_HPO_4_, 2.9 g NH_4_ acetate, 1 g MgSO_4_·7H_2_O, 0.5 g FeSO_4_·7H_2_O, 0.1 g *p-*aminobenzoic acid, 60 g glucose, and 15 g microbial agar. To create anaerobic conditions, media were purged with N_2_. Fermentation tests were performed in vials, in parallel duplicates from the same preculture. Data shown in Tables 1 and 2 are mean results of the duplicate fermentations.

**Table 1:** Fermentation parameters of cultures of *C. beijerinckii* wild type and mutants grown on synthetic medium

Strain		[Glucose consumed] (g/L)	pH at end time	[Solvent] (g/L)				Yield IBEA/GLc	[Acid] (g/L)	
				Ethanol	Acetone	Isopropanol (I)	Butanol (B)		Acetate	Butyrate
Wild type		18^*^	5.8	0.24^^^	0.09^^^	1.54	5.69	42 %	1.11^*^	0.77^^^
EBB	2	16	5.6	0.25^^^	0.08	1.63	5.26	45 %	1.25	0.62
	4	16	6.0	0.29	0.10	1.65	4.90	43 %	1.31	0.48
	9	22	6.0	0.14	0.06	1.7	6.75	39 %	0.79	0.39
MBB	2	20	5.8	0.17^*^	0.09	1.80	4.55	33 %	1.28	0.55
	3	22	6.1	0.11	0.04^*^	1.69^^^	4.93	31 %	0.99^*^	0.24^^^
ISO75	2	22	6.1	0.10	0.04	1.60^^^	5.05	31 %	0.91	0.38
	4	19	5.9	0.31	0.12	1.66	5.51	40 %	1.22	0.52
	10	17	6.0	0.25	0.11	1.58	4.73	39 %	1.25	0.32
ISO50	2	18	6.0	0.31	0.13	2.06	5.99	47 %	1.05	0.29
	10	23^*^	5.1	0.23	0.00	1.66	4.31^^^	27 %	0.96	1.81^*^
AA		18	5.0	0.05	0.88	0.1	2.9	22 %	0.9	4.3

Fermentations of 48 h were carried out in duplicate in serum flasks inoculated with 2 % (*v*/*v*) overnight preculture. Cultures of mutants with higher final isopropanol concentrations are highlighted. The data have standard deviation (SD) <10 % except those with (^^^) 10–20 % SD and (^*^) 20–35 % SD

**Table 2:** Fermentation parameters of cultures of *C. beijerinckii* shuffled strains grown on synthetic medium

Strain	[Glucose consumed] (g/L)	[Solvents] (g/L)				I/B	[Acids] (g/L)		Parent strain(s)
		Ethanol	Acetone	Isopropanol (I)	Butanol (B)		Acetate	Butyrate	
Round 1 (F1)									
F1.B	30	0.84	1.27	**1.90**	9.95	0.19	1.28	0.26	EBB9
F1.C	22	0.28	0.13	**1.89**	6.90	0.27	1.06	0.40	EBB4, EBB9
F1.F	19	0.17	0.12	**1.90**	5.30	0.36	1.01	0.48^**^**^	ISO75 4
F1.K	14	0.13 ^**^**^	0.08^**^**^	1.62	3.82	0.42 ^**^**^	1.06	0.49^**^**^	ISO754,ISO75 10
F1.Q	22	0.21	0.10	1.47	5.23	0.28	1.09	0.24	MBB2, MBB3
F1.X	22	0.15 ^*^	0.04^**^**^	1.33	4.99	0.27	0.94	0.54	MBB2, MBB3, ISO50 2
Round 2 (F2)									
BB 45	25	0.25	0.08	1.83	6.10	0.30	0.80	0.20	F1.B
FF 45	24	0.21^*^	0.10^*^	1.81	5.88	0.31	0.74	0.31^**^**^	F1.F
KK 45	23	0.20	0.09	1.53	5.46	0.28	1.11	0.68	F1.K
KK 50	22	0.22	0.08	**1.81**	6.24	0.29	0.84	0.66	
BF 45	24	0.21^**^**^	0.08^**^**^	1.86	5.89	0.32	0.83	0.19	F1.B, F1.F
BF 50	23	0.22	0.07^**^**^	1.59	4.69	0.34	1.02	0.99	
BK 45	23	0.22	0.06	1.40	5.01	0.28	0.93	0.66	F1.B, F1.K
BK 50	21	0.19^*^	0.06^*^	**1.90**	5.75	0.33	1.03 ^^^	0.60	

Fermentation was performed in duplicate in serum flasks inoculated with 2 % (*v*/*v*) overnight preculture. Data are given as the average of two fermentations. Isopropanol and I/B of some strains are depicted in bold if the values were higher than those of parent strains. The data have standard deviation (SD) of <10 % except those with (^^^) 10–20 % SD and (^*^) 20–35 % SD

*I/B* isopropanol-butanol ratio

For screening purposes, small-scale fermentations were performed in serum bottles containing 50 mL Gapes medium. Bottles were inoculated with 2 % (*v*/*v*) overnight preculture of cells and cultivated for 2–4 days at 37 °C without agitation or pH control. In order to prevent overpressure, a pressure relief valve system is punctured through the rubber stopper used to seal the vials. Samples were taken during fermentation and stored at −20 °C until further use.

### NTG treatment of *C. beijerinckii* DSM6423

N-Methyl-N-nitro-N-nitrosoguanidine (NTG) treatment was performed as described previously (Otte et al. 2009). Ten-milliliter aliquots of early exponential phase cells, optical density (OD) <0.6, were treated with 50 μg/mL NTG for at least an hour at 37 °C. Cells were washed twice in an equal volume of anaerobic potassium phosphate buffer (pH 6.6), resuspended in fresh mCGM medium, and regenerated for at least an hour at 37 °C before spreading on selective plates. Selective plates were prepared with mCGM medium supplemented, as indicated in the text, with 35–40 g/L isopropanol, 1.77 mL/L (ethyl or methyl) bromobutyrate, or 23.8 mL/L allyl alcohol. Thirty-five putatively mutated colonies were isolated in total after growth on selective plates.

### Genome shuffling

Genome shuffling was carried out as previously described (Gao et al. 2012). Cultures of the mutant strains at mid-exponential growth phase (O.D. approx. 0.5–0.6) were centrifuged, and the cell pellet was washed with SMM buffer (0.5 M sucrose, 20 mM sodium maleate monohydrate, and 20 mM MgCl_2_, pH 6.5) and then treated with lysozyme (15 mg/mL in SMM, containing 1 g cysteine/L and 1 g glutathione/L) at 37 °C for 1 h to produce protoplasts. After checking for their presence under the microscope, protoplasts were diluted in SMM (added 1 g cysteine/L and 1 g glutathione/L) and harvested by centrifugation at 4000*g* for 5 min. Protoplasts from different populations were mixed in SMM (containing 1 g cysteine/L, 1 g glutathione/L, 30 % PEG 4000, and 50 mM CaCl_2_) at 37 °C for 20 min to induce fusion. Protoplasts were harvested by centrifugation at 4000*g* for 5 min and resuspended in mCGM medium. The fused protoplasts were spread out on mCGM medium agar plates, and anaerobic cultured at 37 °C for at least 24 h. The resulting colonies were transferred to mCGM agar plates (containing 40–50 g/L isopropanol) and incubated anaerobically at 37 °C for at least 24 h. Then, the strains, with increased isopropanol tolerance, were screened for IBE production in liquid medium. The identified strains with higher performances were used for subsequent rounds of genome shuffling, which were carried out by repeating the protoplast fusion protocol described above.

### Sporulation and toxicity tests

To obtain spores, Gapes agar plates were inoculated with 0.2 mL of *C. beijerinckii* cultures and plates were stored in an anaerobic jar. An anaerobic generator (Oxoid AnaeroGen, Thermo Scientific) and anaerobic indicator (Oxoid Resazurin, Thermo Scientific) were placed inside the jar to maintain and check for anaerobic conditions. The plates were incubated at 37 °C for 3 weeks. The spores were harvested by adding 4–5 mL sterile physiological water (0.9 % *w*/*v* NaCl) and scraping the plates. The spore suspensions were collected, glycerol (20 %, *v*/v) was added, and spores were stored at −20 or −80 °C for longer-term storage.

Spore activation was done by heat-shocking the clostridial spore suspensions in a boiling water bath for 1 min. For the toxicity tests, successfully sporulating spore suspensions were inoculated (0.6–1.0 % *v*/*v* depending on the amount of spores) into 50-mL fresh mCGM medium. The cultures were grown overnight at 37 °C without shaking (OD600 = 2; cells = rod-shaped and very motile). The OD600 was measured using Ultrospec 2000 (Pharmacia Biotech). A droplet of the preculture (5 μL) was dropped onto freshly poured mCGM agar plates containing 30 to 50 g/L isopropanol.

### Analytical methods

Metabolites were determined in clear supernatants of samples taken from the fermentation. Sugars, solvents, and organic acids were determined by HPLC using a gel permeation/size exclusion column (Shodex Ionpack KC-811) coupled to a refractometer and UV detector as described earlier (Collas et al. 2012).

## Results

### Tolerance tests

The tolerance of *C. beijerinckii* to isopropanol, bromobutyrate, and allyl alcohol was determined using agar plate assays (data not shown). When grown on agar plates supplemented with isopropanol ranging from 0 to 50 g/L, growth of wild-type (WT) *C. beijerinckii* is hindered by inhibition of 35 g/L isopropanol. Growth inhibition was observed on plates containing 0.5 mL/L ethyl or methyl bromobutyrate and 8 mL/L allyl alcohol. Selective compounds were chosen according to previous studies and based on literature. Treatment with allyl alcohol has been reported to result specifically in mutants of *C. acetobutylicum* defective in butanol synthesis (Dürre et al. 1986). Bromobutyrate interferes with the final fermentation profiles mainly by reducing butyraldehyde and butanol dehydrogenase activities in *C. acetobutylicum* ATCC824 (Clark et al. 1989).

### Mutagenesis of *C. beijerinckii* DSM6423

Genome shuffling is a recursive method enabling accelerated evolution toward a desired phenotype. It therefore requires a large diversity of starting phenotypes. New phenotypes as compared to the wild type are often obtained through a preliminary mutagenesis step (Zhang et al. 2002). Early exponential phase *C. beijerinckii* cells were exposed to NTG. Fifty micrograms per milliliter of NTG was employed for the selection on the different toxic substrates, allyl alcohol, and ethyl or methyl bromobutyrate, but two NTG concentrations (50 and 75 μg/mL) were used for the isopropanol selectivity tests. To distinguish between these two concentrations, final mutant strains were labeled ISO50 and ISO75, for 50 and 75 μg/mL NTG used for mutagenesis, respectively. After mutagenesis and selection, 36 mutant strains were successfully isolated.

Two series of 10 isopropanol-tolerant colonies, “ISO50” and “ISO75,” were screened for their fermentation performance in 50-mL fermentation vials after selection on plates containing 40 g/L isopropanol. The ISO50 2 mutant strain showed the highest improvement in solvent production and was therefore one of the strains selected for genome shuffling (Table 1). ISO75 6 was also selected due to its lower butanol production. Fifteen bromobutyrate-, 10 ethyl-bromobutyrate “EBB”-, and 5 methyl-bromobutyrate “MBB”-tolerant strains were screened after selection, as well as an allyl alcohol-resistant one. EBB 9 was selected for shuffling as it showed greatly improved performances, although essentially in butanol production. One allyl alcohol-resistant strain, labeled “AA,” was also isolated. This strain showed almost no solvent production which corresponds to the same phenotype as the allyl alcohol mutant strain generated from *C. acetobutylicum* ATCC824 (Dürre et al. 1986). This result confirms the possibility to use allyl alcohol to generate mutants defective in butanol synthesis in *Clostridium* strains. Such a strain is of potential interest for future experiments which may involve transformation and overexpression of genes involved in the isopropanol production pathway with the goal of obtaining a selectively and exclusively isopropanol-producing strain.

Among the whole NTG strain collection, a total of 10 mutants produced more isopropanol than the WT after 48 h of fermentation (Table 1). Among the mutants, MBB 2 and ISO50 2 produced the highest levels of isopropanol with concentrations of 1.80 and 2.06 g/L, respectively, which represent increases of 17 and 33 % compared to the WT (1.54 g/L).

Additionally, butanol production looks similar or even slightly lower in the mutants as compared to the wild type, the mutants therefore showing better selectivity for isopropanol production. Interestingly, the remaining total butyrate amount in the improved mutants is systematically lower than in the wild type, suggesting an increased capacity to assimilate the butyrate produced. Finally, seven mutants, EBB 4, EBB 9, MBB 2, MBB 3, ISO75 4, ISO75 10, and ISO50 2, were selected to be parent strains to perform the genome shuffling steps.

### Genome shuffling based on isopropanol tolerance

**Fig. 1:**
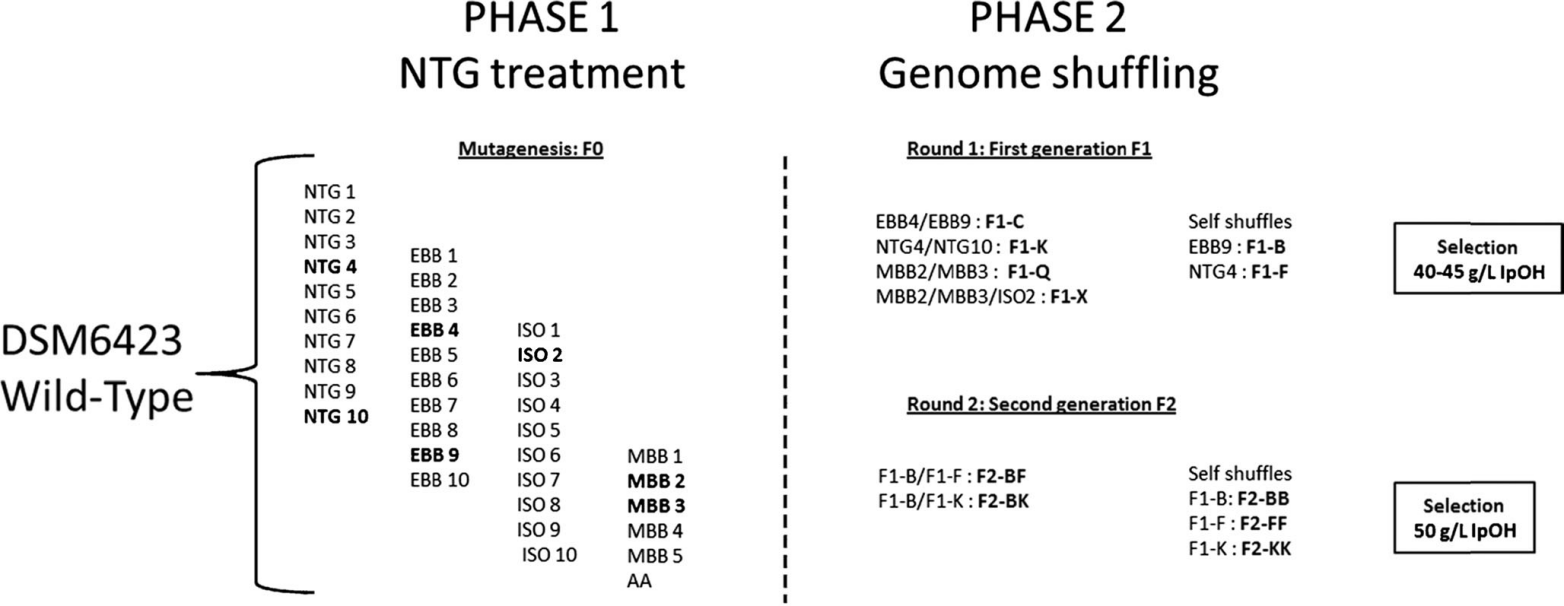
Strains selected for genome shuffling. In the phase 1, strains in *bold* correspond to the selected strains to carry out genome shuffling steps on. First- and second-generation shuffled strains are indicated in *bold* next to their parents. Self-shuffles mean that the shuffling protocol was carried out on cells belonging to a unique NTG mutant corresponding to a unique population of cells. Concentrations of isopropanol used for selection of mutants on plates at each generation is indicated

Two rounds of genome shuffling were performed using the best NTG mutant strains. Several combinations were tested in order to improve the robustness of the IBE strains and their isopropanol tolerance as an easy way to select for putatively enhanced solvent producers. After each step of genome shuffling, potentially enhanced strains were selected based on an improved tolerance toward isopropanol as compared to the parent strains (Fig. 1).

Genome shuffling aims at strengthening mutated strains by improving evolution toward a chosen phenotype, in this case isopropanol tolerance and production. Random mutagenesis usually causes multiple mutations in the strains, some of which might be unwanted or deleterious. Genome shuffling allows recombination of genomes between populations, therefore getting rid of unnecessary mutations while maintaining the interesting ones by maintaining a selective pressure at each step.

**Fig. 2:**
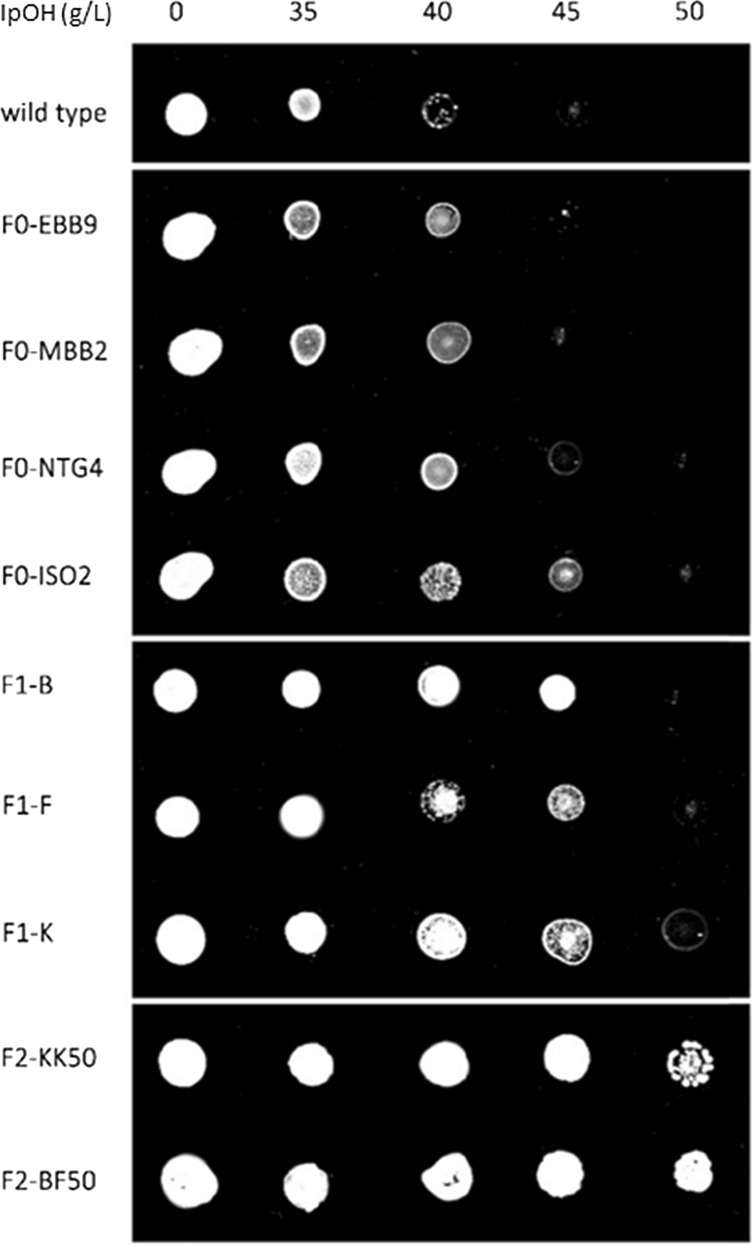
Isopropanol toxicity test plate assay of *Clostridium beijerinckii* NRRL B593 wild type and mutants. *F0 strains* are strains resulting from chemical mutagenesis. *F1 strains* correspond to strains obtained after the first round of genome shuffling. *F2 strains* correspond to the strains obtained after the second round of genome shuffling. mCGM plates containing 0, 35, 40, 45, and 50 g/L isopropanol were used for growth of the colonies. Plates were incubated at 37 °C for 48 h

The shuffling was performed on mutants EBB 9, EBB 4, ISO75 4, ISO75 10, MBB 2, MBB 3, and ISO50 2. Selection was performed on agar plates containing 40–45 and 50 g/L isopropanol for the first and second rounds of shuffling, respectively. The first generation of shuffled strains was isolated on plates containing up to 45 g/L isopropanol as compared to the parent mutants tolerant up to 40 g/L, with an exception made for ISO50 2 tolerant to 45 g/L isopropanol and the initial wild-type DSM6423 which shows growth restriction over 35 g/L. Selection of further improved cell lines was performed on agar plates containing up to 50 g/L isopropanol for the second generation of shuffled strains. Mutations in organisms are not necessarily always stable (Matsubara-Nakano et al. 1980). Therefore, the most interesting mutants from each step of the mutagenesis and shuffling strategy were put to sporulate on Gapes agar. After 3 weeks, spores were activated by heat shock and tolerance tests were performed by plating on selective plates containing isopropanol in order to confirm the isopropanol tolerance improvement (Fig. 2). This isopropanol toxicity test was carried out to confirm the mutant stability post-sporulation, whether they maintained their phenotype toward isopropanol tolerance or not. The wild-type strain and mutants from the mutagenesis step (F0), as well as first (F1) and second (F2) rounds of genome shuffling, were selected as representatives. Growth of the wild type is significantly inhibited at 40 g/L isopropanol, while that of mutant strains (F0) is inhibited at higher concentrations (45 or 50 g/L for ISO50 2). In the case of first-round fusion strains (F1), their growth was not greatly affected at 45 g/L isopropanol. Moreover, the second-round fusants (F2) were able to grow at 50 g/L, unlike any of the other mutants (Fig. 2). This experiment aims to confirm the stability of the selected strains for their respective tolerance levels toward isopropanol post-sporulation. Although most of the strains still showed the improved phenotype, some of the second-generation cross-shuffles lost their tolerance phenotype (data not shown). It is therefore advised to check strain stability over multiple generations (Gao et al. 2012).

### Fermentation profiles of the improved strains

Final improved strains still able to grow on their respective selective plates were tested for their fermentation profile (Table 2). Two rounds of shuffling were performed in total. The strains F1.B and F1.F, resulting from the first generation of shuffles, are the self-shuffled descendants of EBB 9 and ISO75 4, respectively, while F1.C and F1.K correspond to the cross-shuffles between EBB 4 and EBB 9 and ISO75 4 and ISO75 10, respectively. In Table 2, it is shown that F1.B, F1.C, and F1.F produced 1.90 g/L isopropanol after 48 h which is 15 % higher than that of their respective parent strains. Interestingly, F1.B also showed 75 % higher production of butanol than the wild-type strain with 9.95 g/L butanol. Based on the isopropanol and butanol ratio (I/B), F1.F and F1.K showed the highest I/B ratio among the generated shuffled strains, 0.36 and 0.42, respectively. F1.K was especially interesting due to similar isopropanol levels as compared to the wild type but 33 % lower butanol production, improving its selectivity toward the desired product.

Yet, no further improvements in solvent production were observed for fusants F1.Q and X, despite the improvements of the parent strains (MBB2, MBB3, and ISO50 2). The generated fusants produced lower isopropanol titers and I/B ratios. Therefore, the three most promising fusants included F1.B, F1.F, and F1.K which were used for an additional round of genome shuffling.

Five shuffling combinations were carried out including self-shuffling of F1.B, F, and K (as parent strains) and cross-shuffling of F1.B and F and F1.B and K. This second generation of shuffles is designated as F2. The self-shuffled F2.KK and cross-shuffled F2.BF and F2.BK strains were observed to generate colonies on 50-g/L isopropanol plates. Disappointingly, colonies were found only on plates containing 45 g/L of isopropanol for self-shuffles F2.BB and F2.FF. Moreover, the fermentation profile of every F2 strain at 48 h showed no further improvements when compared to F1 strains. The selected F2 strains resulted in lower or similar isopropanol titers and isopropanol-butanol ratios (I/B) when compared to the parent F1 strains.

Nonetheless, a few characteristics can be pointed out. Fusants with higher isopropanol tolerance such as F2.KK50 and F2.BK50 are able to produce more isopropanol than their respective fusants with lower isopropanol tolerance, F2.KK45 and F2.BK45, respectively. F2.KK50 resulted in 18 % more isopropanol than F2.KK45, while F2.BK50 produced 36 % more than F2.BK45. This improvement tends to confirm the existence of a link between increased tolerance to an end product and its production capabilities. Nevertheless, a similar characteristic was not displayed by F2.BF50, since it excreted 1.59 g/L isopropanol which was 15 % lower than F2.BF45, but could be explained by a delay in acid reassimilation as the concentration of butyrate in F2.BF50 was twice higher than in F2.BF45.

**Fig. 3:**
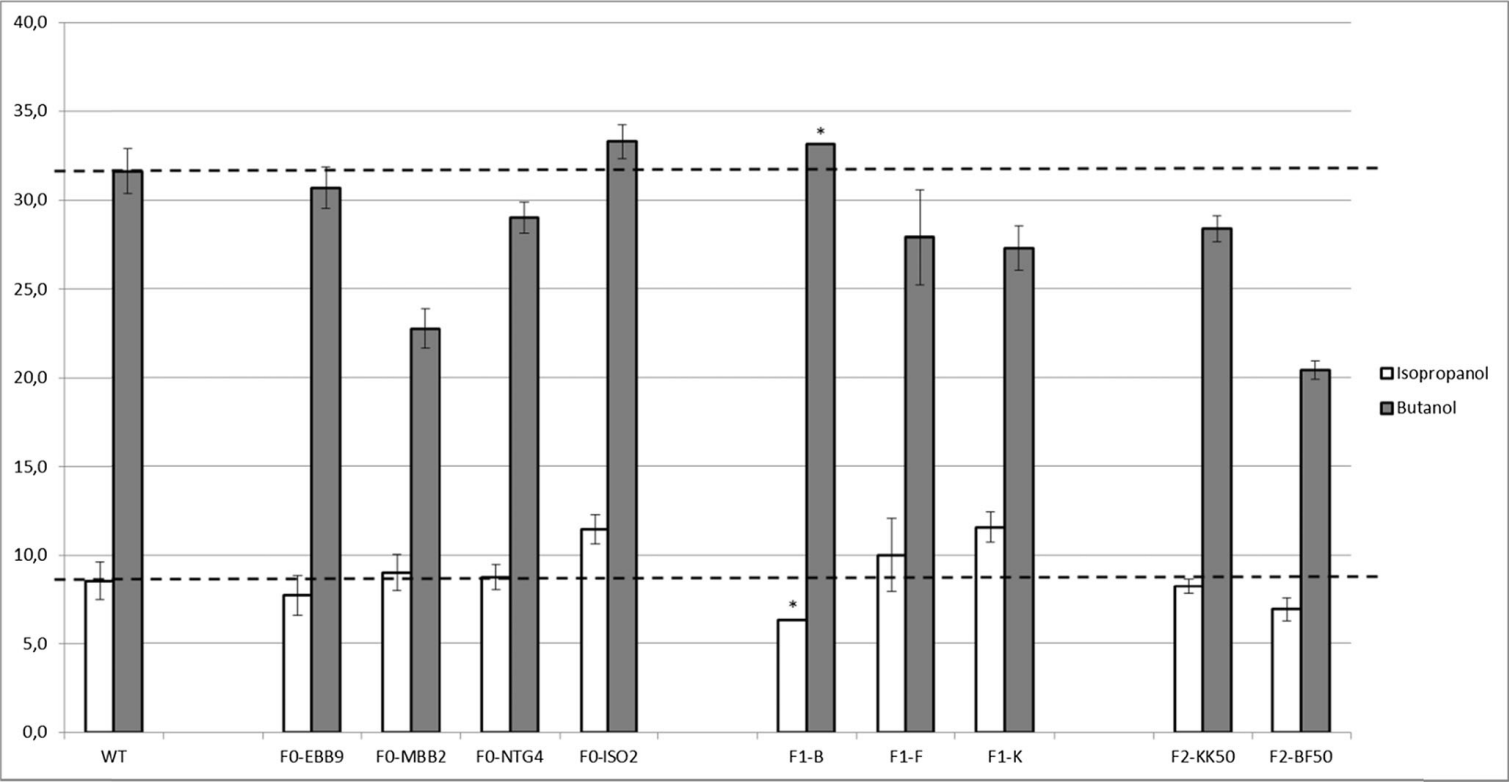
Isopropanol and butanol yields of wild-type and selected mutant strains. Isopropanol and butanol production as compared to the amount of sugar consumed (in %)

The first shuffled strains showing increased solvent production capacities were those coming from the first generation (F1) of self-shuffling performed on EBB9 and ISO75 4. However, a higher tolerance was not systematically linked to a higher solvent production and only the best performing strains were selected for the second round of shuffling. The mutant strains resulting from this second shuffle (F2), although tolerant to higher amounts of solvents, were not characterized by an increase of their solvent production capacities by comparison to their parents of the first generation. This was confirmed by the study of isopropanol and butanol yields (Fig. 3). When using sugar consumption to normalize solvent production, it is observed that the initial mutants from mutagenesis alone (F0), although tolerant to 40 g/L isopropanol, do not have improved yields. An exception is ISO50 2, but this initial mutant was also characterized by a higher tolerance compared to its pairs. First-generation strains F1.F and F1.K showed an improvement in isopropanol selectivity, producing more IpOH together with lower amounts of butanol. But the second-generation F2 strains showed no further improvement in yields, although they were characterized by a higher tolerance.

Representative strains were deposited in the Pasteur Institute strain collection. Initial mutants were EBB9 (as CNCM I-4985), F2.BF50 (as CNCM I-5027), F2.BK50 (as CNCM I-5028), and F2.KK50 (as CNCM I-5029).

## Discussion

Genome shuffling is an approach which helps accelerate the evolutionary process initiated with random mutagenesis by recursive genetic modifications. Thus, it requires a diversified population of mutants showing improvements in the desired phenotype as a starting point (Gao et al. 2012). In our study, isopropanol-tolerant mutants of *C. beijerinckii* DSM 6423 have been produced by chemical mutagenesis. These mutants were subjected to genome shuffling rounds, and the new strains were screened for altered fermentation profiles in comparison to the WT. Out of the 36 initial mutants obtained, 10 showed improved isopropanol production (Table 1). This might be due to the ability of mutants to reassimilate acetate and butyrate better than the WT as suggested by the low levels of these acids detected in the culture broths. Earlier studies on acid reassimilation show that the acid uptake which occurs during solventogenesis is directly coupled to the formation of acetone or isopropanol (Hartmanis et al. 1984; Wang et al. 2012).The remaining 26 mutants showed lower isopropanol production and much higher acid production levels (data not shown). These observations strengthen the role of acid reassimilation in isopropanol production in the *C. beijerinckii* DSM 6423 mutants. The allyl alcohol mutant (AA) showed notably low butanol production and only traces of other solvents concomitant with a possible impairment in butyrate reassimilation suggested by high residual quantities of this acid (Table 1). Previously, similar results have been observed in *C. acetobutylicum* allyl alcohol-resistant mutants, which also showed low butanol production (Dürre et al. 1986). In *C. acetobutylicum* ATCC 824, production of acetone and ethanol was unaffected as investigations revealed that mutants showed very low activity of butyraldehyde dehydrogenase compared to the WT (Dürre et al. 1986). In *C. beijerinckii* DSM 6423, the mutation obtained also seems to affect isopropanol production. Allyl alcohol acts as a suicide substrate which is oxidized into toxic aldehyde, acrolein, by dehydrogenases. Mutations leading to resistance can therefore also affect the secondary alcohol dehydrogenase allowing the DSM 6423 characteristic production of isopropanol from acetone (Ismaiel et al. 1993). The allyl alcohol mutant and the low solvent-producing mutants are considered interesting since these strains can potentially be used as starting strains to generate isopropanol-only producers by reintroducing the isopropanol-related genes and overexpressing them specifically. This approach could increase the level of selectivity for isopropanol production which would allow a more efficient and cheap purification process downstream for industrial purposes.

As isopropanol is the desired end product, a final solvent mix with less by-products would facilitate the separation steps downstream and a strain showing increased selectivity for isopropanol production is therefore of great interest as well.

Several published studies indicate that the genome shuffling approach has been successfully applied to significantly increase the product yield of numerous organisms including *Clostridia*. For example, successful improvement of acetone, butanol, and ethanol production was achieved in *C. acetobutylicum* CICC 8012 (Gao et al. 2012). Genome shuffling is also applicable to other organisms such as yeast. A practical genome shuffling procedure was developed and successfully applied to the nonconventional yeast *Pichia anomala* to increase sugar alcohol production (Zhang et al. 2015). Interspecies shuffling is also possible as demonstrated by the development of a thermostable *Clostridia* resulting in protoplast fusion between mesophilic and thermophilic species and leading to an ability to produce enzymes at higher temperatures (Begum and Dahman 2015).

As observed during the toxicity test (Fig. 2), fusion strains from the first (F1) and second (F2) rounds of genome shuffling were tolerant to higher levels of isopropanol when cultivated on agar plates, 45 and 50 g/L, respectively, as compared to the WT tolerating 35 g/L. This study showed that genome shuffling is an effective way of generating strains with enhanced traits, in this case improved isopropanol tolerance. End product toxicity is being considered as one of the major drawbacks to solvent-producing capabilities in *Clostridium* (Heluane et al. 2011), and in our case, a higher isopropanol tolerance should allow enhanced production of isopropanol. This hypothesis is supported by the current study as three F1 strains—including F1.C, and F1.F—produced 22 % more isopropanol than the WT. However, increased tolerance is not always accompanied by improved production as illustrated by the F2 strains. Also, final isopropanol concentrations are far from the observed tolerance limit. Increased production might therefore not be directly linked to isopropanol tolerance, although it is still an easy and efficient way of selecting potentially improved strains. Among the fusion strains obtained, F1.F was quite interesting since it also showed a 33 % higher isopropanol-butanol ratio than the WT. This ratio is an attractive parameter since it is directly linked to product selectivity, which would allow easier isopropanol purification downstream on an industrial scale.

The F1.B mutant also displayed an interesting profile with 69 % more total solvents than the WT. F1.B especially stood out within the generated fusants for its butanol production of 9.95 g/L, which is almost two times higher than that of the WT grown under the same conditions. F1.B seems to have “gained” efficiency in butyrate reassimilation toward butanol production since its butyrate level is lower than that of other strains. This is in accordance with the fact that its parent strain, EBB 9, already showed improved butanol production which enhanced even more after self-shuffling. It is to be noted though that solvent yield studies tend to minimize fermentation production capability differences between the WT and mutants, since, as shown in Fig. 3, when normalized through glucose consumption, most mutants tend to show similar production capacities as the WT, with a few exceptions. This illustrates the limits of genome shuffling. Selection was based on isopropanol tolerance which is a multi-factorial phenotype, not necessarily accompanied by improved production.

Interestingly enough, it was observed that self-shuffled strains showed better performances than true shuffles when looking at solvent production levels. This phenomenon might be explained by the fact that the disparate parent strains probably carry mutations in different genetic loci and that, when shuffled together, these mutations have a chance to cure each other out, bringing the cells to revert back toward the initial phenotype. Mixed populations are genetically more varied, but due to screening limitations, this variability can be detrimental when trying to randomly isolate an improved strain for a specific aspect, as the probability for a strain to acquire several potentially detrimental mutations in some aspect is high.

When shuffling the same population, holding the same mutations, strains can acquire new traits by recombination, duplication, or deletion events, but chances to maintain the newly acquired mutations are higher as compared to heterogenous shuffling where mutations can be cured between the different mutants. It was also observed that no further improvement was visible after the first round of self-shuffling, the diversity of children cells being higher when mixing different populations. This seems to illustrate the limitations of consanguine lineages in nature.

Despite the relative success of the first shuffling step, no further improvement in solvent production capabilities was observed following the second round of genome shuffling. Even though the strains F2.KK50, F2.BF50, and F2.BK50 tolerated higher isopropanol levels than their parents, they excreted similar amounts of solvents. This demonstrates that enhanced tolerance is not necessarily accompanied by higher production capabilities.

One of the bottlenecks of the genome shuffling approach described here is finding a large-scale, high-throughput screening method in order to test a high number of resulting fusion strains after each round of genome shuffling. It may be difficult to screen large populations for increased product outputs, especially if the detection of the product of interest, isopropanol, requires culturing and no simple on-line methods are available for it, as is the case for butanol (Scheel and Lütke-Eversloh 2013; Patnaik 2008). In addition, an effective strain selection technique allowing to weed out the nonfused protoplasts from the library would be helpful. Thus, in possible future studies, subsequent genome shuffling can be done by applying a wider screening of the fusant strains in order to maximize chances of isolating strains showing improved fermentation characteristics.

Identifying mutations, possible SNPs, and or genetic reorganizations and recombinations which occurred during the sequential improvement described in this study can potentially be of great interest to identify genes involved in clostridial tolerance to end products and/or solvent production. For example, the mutants might have acquired a phenotype resulting in a higher expression of the *ctfAB* genes encoding CoA transferases, enzymes involved in the reassimilation of acids, and catalyzing the first steps of their conversion into solvents (Berezina et al. 2012)*.* Past studies show that overexpression of *ctfAB* leads to better acid reassimilation and 21–31 % higher butanol production in *Clostridium tyrobutyricum* (Yu et al. 2011). To confirm this hypothesis, analysis of gene expression levels in the mutants would also have to be conducted in future studies.

This study describes the application of genome shuffling for the improvement of IBE production in *C. beijerinckii*. Isolation of initial NTG mutants by various selective compounds and further selection of shuffled strains for enhanced isopropanol tolerance contributed to the screening of strains with an increased isopropanol tolerance and improved IBE production capacities. Results described in this study suggest that genome shuffling can be a useful tool to modify the product tolerance in *C. beijerinckii* and possibly other organisms. Strains from the second round of genome shuffling were tolerant up to 50 g/L isopropanol, with 43 % improvement compared to the WT which shows growth inhibition at 35 g/L isopropanol in the medium. Yet, the enhanced tolerance was not systematically accompanied by higher solvent production capabilities as further improvement in isopropanol production was not observed in strains from the second round of shuffling. Despite that, improvements could still be obtained from the first round, and best strains were able to produce up to 23 % more isopropanol and 21 % more butanol than the WT strain. Our results confirm that genome shuffling can tremendously improve the phenotype of the natural IBE-producing strain *C. beijerinckii*.
